# Preliminary Evidence That Circadian Alignment Predicts Neural Response to Monetary Reward in Late Adolescent Drinkers

**DOI:** 10.3389/fnins.2022.803349

**Published:** 2022-02-16

**Authors:** Brant P. Hasler, Jessica L. Graves, Adriane M. Soehner, Meredith L. Wallace, Duncan B. Clark

**Affiliations:** Department of Psychiatry, University of Pittsburgh School of Medicine, Pittsburgh, PA, United States

**Keywords:** adolescence, sleep, circadian rhythms, circadian misalignment, reward, alcohol, fMRI

## Abstract

**Background:**

Robust evidence links sleep and circadian rhythm disturbances to alcohol use and alcohol-related problems, with a growing literature implicating reward-related mechanisms. However, the extant literature has been limited by cross-sectional designs, self-report or behavioral proxies for circadian timing, and samples without substantive alcohol use. Here, we employed objective measures of sleep and circadian rhythms, and an intensive prospective design, to assess whether circadian alignment predicts the neural response to reward in a sample of late adolescents reporting regular alcohol use.

**Methods:**

Participants included 31 late adolescents (18–22 y/o; 19 female participants) reporting weekly alcohol use. Participants completed a 14-day protocol including pre- and post-weekend (Thursday and Sunday) circadian phase assessments *via* the dim light melatonin onset (DLMO), in counterbalanced order. Sleep-wake timing was assessed *via* actigraphy. Circadian alignment was operationalized as the DLMO-midsleep interval; secondary analyses considered social jet lag based on weekday-weekend differences in midsleep or DLMO. Neural response to reward (anticipation and outcome) was assessed *via* a monetary reward fMRI task (Friday and Monday scans). Alcohol use was assessed at baseline and *via* ecological momentary assessment. Mean BOLD signal was extracted from two regions-of-interest (striatum and medial prefrontal cortex, mPFC) for analyses in regression models, accounting for age, sex, racial identity, and scan order.

**Results:**

In primary analyses, shorter DLMO-midsleep intervals (i.e., greater misalignment) on Thursday predicted lower striatal and mPFC responses to anticipated reward, but not reward outcome, on Friday. Lower neural (striatum and mPFC) responses to anticipated reward on Friday correlated with more binge-drinking episodes at baseline, but were not associated with alcohol use in the post-scan weekend. In secondary analyses, greater social jet lag (particularly larger weekend delays in midsleep or DLMO) was associated with lower neural responses to reward anticipation on Monday.

**Conclusion:**

Findings provide preliminary evidence of proximal associations between objectively determined circadian alignment and the neural response to anticipated monetary reward, which is linked in turn to patterns of problematic drinking. Replication in a larger sample and experimental designs will be important next steps to determining the extent to which circadian misalignment influences risk for alcohol involvement *via* alterations in reward function.

## Introduction

Robust evidence links sleep and circadian rhythms to alcohol use and alcohol-related problems, including accumulating longitudinal evidence indicating that sleep/circadian disturbances prospectively predict increases in alcohol use and problems (Hasler and Clark, 2013; Koob and Colrain, 2020; Troxel et al., 2021). Although the mechanisms by which sleep/circadian factors influence alcohol use remain unclear, a number of viable contenders have emerged, including alterations in reward function (Hasler and Pedersen, 2020; Laniepce et al., 2021). Altered reward function is central to prominent addiction theories. However, theories and research findings differ in their emphases and observations on reward anticipation vs. outcomes, on the direction of effects (i.e., hypo- vs. hyper-responsivity) and the role of impaired cognitive control (Berridge and Robinson, 2016; Bjork, 2020). During adolescence, circadian misalignment—a temporal mismatch between behavioral sleep/wake schedules and the circadian clock—may exacerbate risk for alcohol use by altering the neural reward processing (Hasler and Clark, 2013).

A range of sleep and circadian characteristics associated with circadian misalignment have been linked to alcohol use and related problems (Wong et al., 2015; Hasler et al., 2017b; Haynie et al., 2017; Troxel et al., 2021). Circadian misalignment during adolescence primarily occurs due to a mismatch between adolescents’ tendency toward late sleep and circadian timing and the academic and/or work schedules that are timed too early (Crowley et al., 2018). Misalignment is worse for those with tendencies for later sleep/circadian timing (Duffy et al., 2001; Mongrain et al., 2006), and can result, in turn, in difficulty falling asleep at night (insomnia), insufficient sleep duration, and daytime sleepiness. Marked differences between sleep timing on school/work days and free days (typically weekends), termed social jet lag, is another manifestation of this misalignment (Wittmann et al., 2006; Crowley et al., 2018). Accordingly, insomnia, shorter sleep duration, and daytime sleepiness, as well as evening circadian preference, late chronotype, and social jet lag, are associated with alcohol use and problems in both cross-sectional and longitudinal datasets (Wong et al., 2015; Hasler et al., 2017b; Haynie et al., 2017; Troxel et al., 2021). Most recently, we reported preliminary evidence that an objective measure of circadian alignment, the phase angle or interval between the dim light melatonin onset (DLMO) and the midpoint of sleep based on actigraphy, was associated with weekend alcohol use in a sample of late adolescent drinkers (Hasler et al., 2019a). Specifically, having a shorter DLMO-midsleep phase angle (consistent with individuals with later circadian phase attempting to sleep at an earlier biological time) was associated with greater alcohol use on the weekend.

Both preclinical and clinical research have demonstrated that reward function is modulated by sleep and circadian factors, with preclinical experimental studies in particular underscoring extensive interactions between circadian and reward pathways in the brain, such as clock gene regulation of dopamine neurotransmission in the nucleus accumbens (Webb et al., 2015; Logan et al., 2018). Evidence from the human literature has relied more on non-experimental designs and proxy measures for circadian timing and/or misalignment. These include studies showing a circadian rhythm in reward activation using a measure based on heart rate in a sample of 18–30 year-olds (Murray et al., 2009), time-of-day effects on a behavioral measure of reward motivation in 18–30 y/o men (Byrne and Murray, 2017), and two independent fMRI studies in healthy late adolescent/young adult samples showing time-of-day effects on the neural response to monetary reward using variants of a card-guessing task (Hasler et al., 2014; Byrne et al., 2017).

Other human fMRI studies have focused on a self-reported circadian preference for eveningness or social jet lag, along with variants of the same card-guessing monetary reward task. The newer variants of this task explicitly include separate anticipation and outcome phases in order to parse the theoretically distinct “wanting” and “liking” aspects of reward (Berridge and Robinson, 2016). In data from a study of late adolescents (age 20–22; all male), evening circadian preference was associated with altered neural response during a monetary reward fMRI task, as well as alcohol outcomes, both cross-sectionally and over a 2-year follow-up (Hasler et al., 2013, 2017a). In the cross-sectional data, evening-types exhibited relatively lower mPFC response during outcome phase of the reward task and higher striatal response during anticipation phase of the reward task linked, respectively, to greater alcohol use and symptoms of alcohol dependence. A somewhat different pattern emerged in the longitudinal data—greater eveningness at age 20 predicted higher mPFC and striatal response to reward outcome, and age 22 mPFC response to reward statistically mediated the association between age 20 eveningness and age 22 symptoms of alcohol dependence. In a separate study of healthy younger (age 12–14) adolescents, greater social jet lag based on actigraphy data was associated with decreased striatal and mPFC response to monetary reward (Hasler et al., 2012). Findings were similar across anticipation and outcome phases of the task, although stronger for the mPFC, and held after accounting for total sleep time. It is important to consider sleep duration, as sleep deprivation has been linked to altered reward function in multiple past fMRI studies (Venkatraman et al., 2007, 2011; Gujar et al., 2011; Mullin et al., 2013). Together, these studies support associations between sleep/circadian rhythms and reward-related brain function, with differences in the direction of neural response (increases or decreases) potentially explained by the divergent sleep/circadian measures and/or developmental differences between the samples.

These mixed findings in terms of directionality (increases or decreases) of reward response, as well as in the specificity of reward phase (anticipation vs. outcome/receipt), have parallels in the addiction literature. As noted above, both directionality and reward phase are theoretically relevant across multiple theories of addiction. However, as recently reviewed by Bjork (2020), the extant neuroimaging data are indeterminate as to whether increased or decreased reactivity in mesolimbic reward circuitry (particularly the striatum) is linked to elevations in risky behavior. With regard to reward phase, theories of risk tend to emphasize anticipatory aspects of reward (reward wanting or seeking), and the incentive-sensitization theory explicitly focuses on changes in anticipatory processes (and underlying dopaminergic neurocircuitry) as the most relevant to risk (Berridge and Robinson, 2016). Few neuroimaging studies have explicitly parsed the anticipation and outcome phases of reward with respect to addiction risk (Tervo-Clemmens et al., 2020). The nature of the reward is likely relevant to interpreting these complex literatures as, based on the incentive-sensitization theory, there may well be a divergent anticipatory response to the monetary reward tasks primarily employed in the sleep/circadian literature (i.e., non-drug rewards) vs. alcohol or other drug-related rewards. Thus, while the preponderance of evidence supports increased striatal response to reward as an indicator of addiction-related risk (Heitzeg et al., 2015; Tervo-Clemmens et al., 2020), there is also evidence that striatal response to non-drug rewards goes down with increasing substance use (Bjork, 2020). Once a substance use disorder (SUD) is present, striatal response may be relatively increased during reward *anticipation* but relatively decreased during reward *outcome* (Luijten et al., 2017).

Although the described studies collectively support an association between sleep/circadian proxies for circadian misalignment and reward-related brain function, their mostly cross-sectional designs preclude determination of a causal effect. With this in mind, we recently reported novel evidence from an experimental manipulation of circadian alignment in healthy 13–17 y/o adolescents that provided the first causal evidence of circadian misalignment impact on human reward function (Hasler et al., 2021). Misalignment was associated with a lower ventral striatal response during the outcome phase of the monetary reward task, after accounting for the prior night’s total sleep time. Interestingly, misalignment was also associated with lower right inferior frontal gyrus reactivity during response inhibition on a Go/No-Go task, which could reflect impairments in impulse control. However, none of the adolescent participants were engaging in regular substance use, precluding examination of whether these alterations in reward function due to misalignment were related to substance use.

Here we attempted to extend the literature using an objective measure of circadian misalignment (DLMO-midsleep), an fMRI monetary reward task, and alcohol use in late adolescent drinkers studied over 8 days. A strength of our study design was its capacity to look at prospective, proximal relationships among the constructs of interest. We examined circadian misalignment (DLMO-midsleep phase angle) at a theoretically relevant time—Thursday evening, when participants would presumably be in the midst of circadian misalignment induced by school/work schedules. We examined reward function during midday on Friday, immediately following the circadian alignment assessment and prior to engaging in any weekend alcohol use. We examined alcohol use over Friday and Saturday nights, the peak nights of alcohol consumption in adolescents. Our primary aim was to examine whether weekday (Thursday) circadian misalignment predicted pre-weekend (Friday) reward function, and whether pre-weekend reward function predicted, in turn, weekend (Sat/Sun) alcohol use. Based on prior findings in late adolescents (Hasler et al., 2013, 2017a), we hypothesized that greater misalignment on Thursday (shorter DLMO-midsleep phase angle) would predict higher striatal and lower mPFC responses during reward anticipation and outcome on Friday, and that these neural responses during reward would predict greater weekend alcohol use. In line with prior work on circadian misalignment (Lewy et al., 2006; Hasler et al., 2019a), our analytical models presumed that misalignment more broadly would be evidenced by deviations in either direction from a “normative” phase angle, but that deviating in the direction of shorter phase angles (typical of delayed circadian timing) would best characterize circadian misalignment associated with altered reward function in this late adolescent sample.

We also implemented secondary analyses addressing whether post-weekend circadian misalignment based on DLMO-midsleep, as well as social jet lag as an alternative and widely studied measure of circadian misalignment, were associated with reward-related brain function. First, our study design included a post-weekend (Sunday) circadian alignment assessment, and Monday monetary reward fMRI scan, allowing us to explore whether circadian misalignment at the close of the weekend predicted reward function at the start of the work/school week. Although we viewed this as less obviously relevant to alcohol use (which occurs to a larger degree on the weekend), it could potentially point to other reward-related outcomes (e.g., anhedonia) that could impact work/school performance and overall weekday functioning. Second, in parallel to our prior manuscript in this sample (Hasler et al., 2019a), secondary analyses also examined whether “classic” social jet lag (based on weekday-weekend differences in actigraphy-based midsleep) and “objective” social jet lag (based on weekday-weekend differences in DLMO), were associated with reward function variations on Friday or Monday.

## Materials and Methods

Participants in the present sample included 31 healthy late adolescent (18–22 y/o, 19 identified as female) alcohol drinkers, all reporting a minimum of one standard drink per week over the past 30 days [assessed *via* a web- or phone-based timeline follow back (TLFB)]. The sample overlaps with those described in a prior manuscript (Hasler et al., 2019a). Participants were recruited *via* flyers and a research registry. They were required to be right-handed to avoid lateralization issues for the neuroimaging component of the study. We recruited adolescents with a range of alcohol use levels, including light (*n* = 3; < 12 drinks/month, no binges), moderate (*n* = 19; 13–28 drinks/month and/or 1–4 binges), and heavy (*n* = 9; > 28 drinks/month and/or 5+ binges) drinking. Written informed consent was obtained for all participants. The study was approved by the Institutional Review Board of the University of Pittsburgh.

Participants were excluded for past month substance use (other than alcohol, cannabis, or nicotine) based on the screening TLFB, or if they reported significant current medical (e.g., liver disease, cardiovascular disorder, head injury with loss of consciousness), or psychiatric (e.g., major depression, schizophrenia, and bipolar disorder) illness. Psychiatric disorders were diagnosed *via* Structured Clinical Interview for DSM-IV (First et al., 2002). Participants were also excluded for any current sleep disorders other than insomnia, as determined *via* a locally developed structured interview for sleep disorders based on DSM-IV and the International Classification of Sleep Disorders, 3rd edition (American Academy of Sleep Medicine, 2014). We included individuals with insomnia given that insomnia was expected to be prevalent among our population of greatest interest—those with regular alcohol use and/or circadian misalignment.

Participants with extreme habitual sleep times (bedtimes later than 2:00 am, rise times later than 10:00 am) or habitual sleep durations (longer than 9 h or shorter than 6 h) were also excluded due to the practical challenges of studying participants with extreme sleep times (e.g., difficulty staffing the sleep lab) and concern over having extreme outliers in a relatively small sample. Finally, participants were excluded if they used medications that interfere with sleep and/or reward function (e.g., hypnotics, antidepressants, anxiolytics, beta blockers, benzodiazepines, and stimulants).

A total of 41 participants were consented for the study. One participant was ruled ineligible due to past month illicit substance use, one was withdrawn by investigators due to poor adherence to study procedures, one withdrew due to illness, and two withdrew due to schedule conflicts. Of the 36 who completed the protocol, three participants were excluded due to missing the fMRI scans, and an additional two participants were excluded due to poor quality fMRI data (see supplementary section “Neuroimaging Preprocessing and Analysis”), resulting in a maximum sample of 31 participants for the present analyses.

### Protocol

#### *In vivo* Assessment

All participants completed a 14-day ecological momentary assessment (EMA) protocol, which was designed to assess their sleep and alcohol use (along with affect, craving, and other measures not discussed here) throughout the day under naturalistic conditions. Participants were permitted to keep an ab libitum sleep schedule on all nights outside of the lab visits. The EMA protocol was administered *via* smartphones; text message prompts were sent six times a day with a link to a secure, browser-based assessment system developed by the Office of Academic Computing in the Department of Psychiatry at the University of Pittsburgh School of Medicine. For the present analyses, we focus on alcohol use as collected *via* self-report from the first morning assessment, which was prompted *via* text at participants’ self-reported habitual rise time. The response window remained open until noon, and participants were incentivized to complete the morning assessment (completion was required to earn that day’s payment).

#### Laboratory Assessment

**FIGURE 1:**
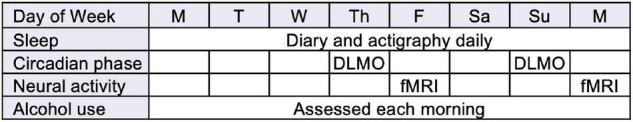
Study design (abbreviated from full 14-day protocol to focus on key assessments; order FM shown).

Participants came into the Sleep and Behavioral Neuroscience Center (SBNC) for two pre-weekend (Thursday/Friday) and post-weekend (Sunday/Monday) overnight visits to assess circadian phase *via* salivary melatonin. Participants were instructed to refrain from alcohol and other drug use for 24 h prior to the visit; a breathalyzer and urine drug screen were used to verify abstinence. The fMRI scans were completed the following day (all scans completed between 9:40 and 12:53, mean = 10:45) in the Magnetic Resonance Research Center. The order of these visits was counter-balanced to address potential task habituation effects during each visit’s fMRI scan; participants were randomly assigned to either a Thursday/Friday-Sunday/Monday (‘FM’) or Sunday/Monday-Thursday/Friday (‘MF’) order. See Figure 1.

### Measures

#### Sleep and Circadian Phase

Our primary measure of sleep-wake patterns was *via* wrist actigraphy, although sleep was also assessed daily in the electronic diary rise time assessment using a standard sleep diary, which includes items corresponding to sleep timing, continuity, and quality. Participants wore an Actiwatch Spectrum Classic (Philips Respironics, Bend, OR, United States) during the entire 14-day study period. On average, actigraphy data was available for 11.70 out of the 14 days (*SD* = 3.26; 82.7% completion rate) across the full sample. Actigraphs recorded activity on medium sensitivity at 1-min epochs. Participants received instructions to press the event recording marker on the watch to indicate when they (a) started to try to fall asleep and (b) when they woke up for the day. These event markers were used to set the start and end of the rest interval. If event markers were not available, participant self-reported bed and wake times from morning sleep diaries were used. If sleep diary data were not available, then a consensus meeting among study personnel was used to determine the rest interval based on changes in activity and light data. After making these adjustments, we derived several actigraphy-based sleep variables for the analyses described below. We calculated midsleep (midpoint between sleep onset and sleep offset) from each of the two nights prior to the night of DLMO assessment were averaged to estimate a person’s midpoint of sleep, a widely used and parsimonious measure of sleep timing. We also calculated total sleep time (TST) based on the interval between sleep onset and sleep offset, minus wake after sleep onset, from the night prior to each fMRI scan to account for sleep restriction due to the DLMO collection and sleeping in the lab. Actigraphy data from the relevant study nights (see below) was not available for three participants; therefore, these participants were excluded from analyses.

Circadian phase was assessed *via* the salivary DLMO (Benloucif et al., 2008) during the Thursday (pre-weekend) and Sunday (post-weekend) overnight laboratory visits. Saliva samples (to assess melatonin) were collected in Salivettes (Sarstedt, Newton, NC, United States) under dim light conditions (<15 lux at any angle of gaze) every half-hour starting 6 h before, and ending 1 h after, each participant’s habitual bedtime. Dim light conditions were instituted starting 1 h before collection and were confirmed each visit using a light meter. The sampling protocol followed standard procedures to control for posture (participants remained seated other than trips to the bathroom) and other possible confounding factors (e.g., no eating/drinking within 10 min of sampling; caffeine, bananas, and chocolate were prohibited throughout the study visit, etc.) (Burgess, 2010). Participants were asked to rinse their mouths with water 10 min prior to each sample if they had eaten or drank. After collection, saliva samples were frozen at −80°C and shipped overnight on dry ice for radioimmunoassaying by Solid Phase, Inc. (Portland, ME, United States) using commercially available kits (ALPCO, Inc., Salem, NH, United States). The DLMO was calculated as the clock time when levels exceeded the mean of three consecutive baseline samples plus twice the standard deviation of those samples (Voultsios et al., 1997); this approach produces DLMO estimates that are closer to the initial rise of melatonin than fixed threshold methods (Molina and Burgess, 2011).

We calculated three measures of circadian alignment. (1) *DLMO-Midsleep Phase Angle*: Our primary, and most direct, measure of circadian alignment was based on the interval between DLMO and midsleep (based on actigraphy from the two nights prior to the DLMO assessment). We focused on DLMO-midsleep from the Thursday night DLMO assessment for our primary analyses but also calculated DLMO-midsleep for the Sunday night DLMO assessment for secondary analyses. Midsleep was based on Tuesday and Wednesday nights for the Thursday DLMO assessment and based on Friday and Saturday night for the Sunday DLMO assessment, thus providing proximal measures of sleep timing that were balanced across the two DLMO assessments. We did not include midsleep from the Thursday or Sunday DLMO assessment nights as it was impacted by DLMO collection procedures that kept participants up an hour later and potentially any other factors typically associated with sleeping in a laboratory environment.

Our two secondary measures of circadian misalignment were indicators of social jet lag. (2) *Social Jet Lag-Midsleep*: The first (weekday-weekend differences in actigraphy-based midsleep, aka “classic” social jet lag) was calculated by subtracting the weekday midsleep (mean Tuesday and Wednesday midsleep prior to the Thursday DLMO) from weekend midsleep (mean Friday and Saturday midsleep prior to the Sunday DLMO). (3) *Social Jet Lag-DLMO*: The second (weekday-weekend differences in DLMO, aka “objective” social jet lag) was calculated by subtracting the Thursday DLMO from the Sunday DLMO.

#### Substance Use

During initial screening, the use of alcohol and other drugs over the past 30 days was assessed *via* Timeline Follow Back (TLFB) (Sobell and Sobell, 1992). Some participants completed the TLFB *via* a web-based format (Sobell et al., 1996; Rueger et al., 2012), which included instructions and a calendar covering the past 30 days that allowed entry of relevant events (e.g., parties) and number of drinks for each day. Other participants completed the TLFB *via* a phone interview with a trained staff member. Following the detailed assessment of alcohol use, participants were also asked “Did you use any drugs other than alcohol in the past 30 days?” with a Yes/No response format. Participants answering “Yes” were asked to list the drugs. Additional lifetime drinking and drug use data are not reported here but are available from the authors upon request. Based on the alcohol TFLB data, *total binge days* (4+ and 5+ drinks on a given day for female and male participants, respectively) over the past 30 days were summed as an indicator of problematic drinking history.

The morning EMA battery assessed alcohol use with the question “How many alcoholic drinks did you have yesterday?” with a drop-down menu allowing responses from 0 to 29. This data was used to calculate *weekend alcohol use* following the Friday fMRI scan, calculated as the sum of self-reported alcohol use across the Friday and Saturday following the Thursday DLMO and Friday fMRI scan assessments.

#### fMRI Monetary Reward Task

This 8-min paradigm is a variation of a task that reliably elicits striatal and mPFC response to anticipation and receipt of monetary reward in adolescents and adults (Forbes et al., 2010, 2014; Hasler et al., 2013), and is sensitive to sleep/circadian factors (Hasler et al., 2012, 2021; Casement et al., 2016). Participants can win or lose money by guessing whether a card’s value is high or low. Participants make a guess *via* button press (high or low; *decision*, 4 s), then view an image of shuffling cards with a yellow arrow indicating the trial type (up for possible win, down for possible loss, both up and down for a mixed win/loss condition, no arrow for a neutral card; *reward anticipation*, 2–6 s), then see the “actual” number on the screen for 500 ms and receive 500-ms feedback (up arrow: win $1; down arrow: lose $0.75; yellow circle for neutral), then view a crosshair for 2–6 s (*reward outcome*). There were 48 trials total and an equal number of trials in each condition. In win trials, participants were told they would win $1 if their guess was correct and there would be no change in earnings if their guess was incorrect. In loss trials, participants were told they would lose $0.75 if their guess was incorrect and there would be no change in earnings if their guess was correct. On neutral trials, participants were told they would neither win nor lose money regardless of whether their guess was correct. Participants were unaware of the fixed outcome probabilities in the paradigm and were led to believe their performance would determine net monetary gain. Each participant was given $25 in earnings. Contrasts for the present analyses included reward anticipation (reward anticipation > neutral) and reward outcome (win > neutral).

### Data Analysis

#### Neuroimaging Preprocessing and Analysis

Neuroimaging data were preprocessed and analyzed using SPM12 (Ashburner et al., 2014) and the Artifact Detection Toolbox^1^ (ART). We used the *rex* toolbox^2^ to extract mean activations (*p* = 1.0) across all voxels from *a priori* regions of interest (ROIs): striatum (bilateral caudate and putamen) and mPFC (Supplementary Figure 1). We converted all extracted mean activations to z-scores in order to enhance interpretability. Full details about task presentation, neuroimaging preprocessing, ROI creation/extraction, and analysis are provided in the Supplementary Material section.

#### Primary Analyses (Circadian Alignment Models)

Multivariable linear regression models were used to estimate the associations between circadian alignment (DLMO-midsleep phase angle) and reward function. We used two different approaches to model potentially non-linear effects of circadian alignment might on brain activity: (1) linear deviation spline models (further referred to as the “deviation” model) and (2) tertile models.

##### Deviation Models

Based on prior work in the mood literature (Lewy et al., 2006; Emens et al., 2009) and our prior work with this sample (Hasler et al., 2019a), we assumed that an ideal or normative DLMO-midsleep phase angle exists, and that deviations from this phase angle would be associated with worse function. Also consistent with the prior manuscripts, we assumed that deviations in the direction of shorter DLMO-midsleep phase angles (relative to larger DLMO-midsleep phase angles) would be more strongly associated with altered reward function. While there is yet to be an agreed-upon clinical cut point for the ideal phase angle, prior work in a sample of 23–53 y/o adults with seasonal affective disorder relied on the mean phase angle (6 h) from a comparison healthy sample (Lewy et al., 2006). For this manuscript in a younger late adolescent sample, we selected the sample median DLMO-midsleep phase angle (median = 6.75 h) to best approximate the “normative” phase angle. This model can be parameterized as:


(1) $$\begin{array}{c} Y=\beta_{0}+\beta_{1}{\left(phaseangle-median\left(phaseangle\right)\right)}^{1-}\\ \;+\beta_{2}{\left(phaseangle-median\left(phaseangle\right)\right)}^{1+}+\\ \;\sum_{3}^{k}\beta_{k}covariate_{k} \end{array}$$

where, (*phaseangle* − median(*phaseangle*))^1−^ is the deviation from the median phase angle if participants are ≤ the median and zero otherwise, and (*phaseangle* − *median*(*phaseangle*))^1+^ is the deviation from the median phase angle if participants are > the median and zero otherwise. $\sum_{3}^{k}\beta_{k}covariate_{k}$represents covariates 3 through *k* in the model. Model (1) is effectively a spline model where the spline is set at the median phase angle and allows to understand how deviations in phase angle outside of this median might differ in their associations with reward function.

##### Tertile Models

Recognizing that the literature has not yet converged around an ideal phase angle, and that indeed, healthy individuals can exhibit some degree of variability in phase angle without clear clinical consequence (Burgess and Fogg, 2008), we also took a more agnostic modeling approach by examining how reward function varied across tertiles of DLMO-midsleep phase angle (i.e, “short” 5.92, “medium” ∈ (5.92, 7.02], “long”7.02h phase angles). This model is parameterized as:


(2) $$\begin{array}{c} Y=\beta_{0}+\beta_{1}I\left(phaseangle\leq shortesttertile\right)+\\ \;\beta_{2}I\left(phaseangle>longesttertile\right)+\sum_{3}^{k}\beta_{k}covariate_{k} \end{array}$$

where, the “medium” tertile group is set as the reference group. In this model we test statistical significance of short vs. medium (_1_) and high vs. medium (β_2_). We also test the overall effect of phase angle on the outcome.

Our primary analyses focused on circadian alignment on Thursday predicting Friday reward function. In models where the DLMO-midsleep variable(s) predicated the fMRI reward outcome, we also re-ran additional models to evaluate if effects remained even after adjusting for total sleep time (TST) on Thursday night to account for any effects related to sleep restriction due to the laboratory protocol.

We also ran secondary analyses examining whether circadian alignment on Sunday predicted Monday reward function.

#### Reward Function Predicting Alcohol Outcomes (Primary Analyses)

For fMRI reward outcomes shown to be sensitive to weekday circadian misalignment in the primary analyses, our *a priori* analyses examined if pre-weekend (Friday) reward function was associated with subsequent weekend drinking, as measured by total alcoholic beverages (drinks) on Friday and Saturday. Secondly, we performed a *post-hoc* analysis estimating the association between fMRI activity and alcohol history, specifically the number of binge days over the last 30 days. Because both alcohol use outcomes are count measures, we used multivariate generalized linear regression with Poisson family distribution. For total weekend alcohol beverages, we included an offset term representing the number of valid weekend observations obtained to estimate incidence rate of weekend drink counts. No offset was required for number of binge days as all participants were observed over the same duration (30-day period).

#### Social Jet Lag Models (Secondary Analyses)

In social jetlag models, we used a similar modeling approach (i.e., linear deviation split and tertile models). However, instead of splitting the deviation term at the median social jetlag, we split at social jetlag = 0 (equation 3). This is due to stronger conceptual and scientific understanding that minimizing the difference between midsleep timing on work/school days and free day would be considered an “ideal” or “healthy” phenotype. Additionally, because of the theoretical understanding behind a “healthy” social jetlag phenotype, we focus on linear deviation split models for this manuscript as opposed to tertile models. This model can be parameterized as:


(3) $$\begin{array}{c} Y=\beta_{0}+\beta_{1}{\left(socialjetlag\right)}^{1-}+\beta_{2}{\left(socialjetlag\right)}^{1+}\\ \;+\sum_{3}^{k}\beta_{k}covariate_{k} \end{array}$$

where, (*social jetlag*)^1−^ is the social jetlag value when social jetlag ≤ zero and zero otherwise, and (*social jetlag*)^1+^ the social jetlag value when social jetlag > zero and zero otherwise. We separately tested the effects “classic” and “objective” social jetlag (see Measures section).

These secondary analyses examined associations between social jet lag measures and fMRI reward outcomes on both Friday and Monday.

#### Covariates, Transformations, and Outliers

All fMRI reward data were z-scored (mean = 0, *SD* = 1), to allow for comparability across models and brain areas. Thus, for all models where reward function is a predictor, coefficients associated with reward function are standardized betas and denoted as β. For all models where reward function is the outcome, coefficients can be interpreted as the SD change in fMRI activity corresponding to a 1-unit change in the independent variable and are denoted as b. Covariates for all models included age, sex, racial identity [White, Black (full or biracial), Asian (full or biracial)]), and scan ordering (Friday or Monday first). Additionally, all key independent and dependent variables were tested to identify any outliers (any values ≥ mean ± 3 SD), and any values identified outliers were set to missing and therefore were excluded from specific models including those dependent or independent variables. This allowed us to retain as much data as possible where relevant.

#### Multiple Comparison Correction

The Benjamini-Hochburg (Benjamini and Hochberg, 1995) false discovery rate procedure was used to adjust for multiple comparisons in the Primary Analyses. We grouped tests based on hypotheses: (1) circadian alignment associated with fMRI activity, and (2) fMRI activity associated with alcohol use. Due to correlation between deviation and tertile models, we separated these model types into their own groups. This resulted in the following grouped tests: (1) for primary fMRI outcomes, deviation short and long coefficient estimate *p*-values [8 tests (2 coefficients × 4 outcomes)], (2) for primary fMRI outcomes, tertile short vs. middle and long vs. middle coefficient estimate *p*-values [8 tests (2 coefficients × 4 outcomes)], and (3) for alcohol use outcomes, fMRI activity beta estimate *p*-values [4 tests (1 beta × 2 brain areas × 2 outcomes)].

## Results

The sample description is shown in Table 1. As described previously (Hasler et al., 2019a), the participants were regular drinkers but otherwise healthy, on average not depressed, excessively sleepy, or reporting poor sleep quality (data not shown here).

**TABLE 1:** Sample characteristics.

	*n*	Mean ± SD	Range
Age	31	21.1 ± 1.2	18 – 22
Sex	31	12 males / 19 females	
Racial identity/ethnicity	31	23 White, 3 Black (full or biracial), 5 Asian-American (full or biracial); 2 Hispanic	
Alcohol use at screening (TLFB)			
Total drinks over past 30 days	31	30.5 ± 30.6	7 – 136
Binge days (5+/4+ drinks) over past 30 days	31	3.2 ± 3.6	0 – 15
Alcohol use during the study weekend (EMA)			
Total drinks over Friday and Saturday	24	4.8 ± 4.4	0 – 12
Actigraphy – midsleep			
Tuesday/Wednesday pre-DLMO	28	4:53 ± 1:17	2:49 – 7:44
Friday/Saturday pre-DLMO	28	5:25 ± 1:01	3:03 – 8:45
Actigraphy – total sleep time (TST)			
Thursday DLMO night	25	6 h 23 m ± 1 h 14 m	3 h 10 m – 8 h 40 m
Sunday DLMO night	28	6 h 32 m ± 1 h 3 m	4 h 5 m – 8 h 13 m
DLMO – Thursday	26	22:09 ± 1:32	18:36 – 01:44
DLMO – Sunday	27	22:08 ± 1:30	18:53 – 01:57
DLMO-midsleep phase angle (hours)			
Thu DLMO-Tuesday/Wednesday midsleep	24	6.75 ± 1.47	4.37 – 9.44
Sun DLMO-Friday/Saturday midsleep	25	7.24 ± 1.68	4.45 – 10.76
Social jet lag-midsleep (Friday/Saturday– Tuesday/Wednesday; hours)	26	0.53 ± 1.33	−1.14 – 4.84
Social jet lag-DLMO (Sunday–Thursday; hours)	26	0.04 ± 0.82	−1.40 – 1.79

*TLFB, timeline follow back; EMA, ecological momentary assessment; DLMO, dim light melatonin onset.*

### Primary Analyses – Does Weekday Circadian Alignment Predict Pre-weekend Reward-Related Brain Function on Friday?

**FIGURE 2:**
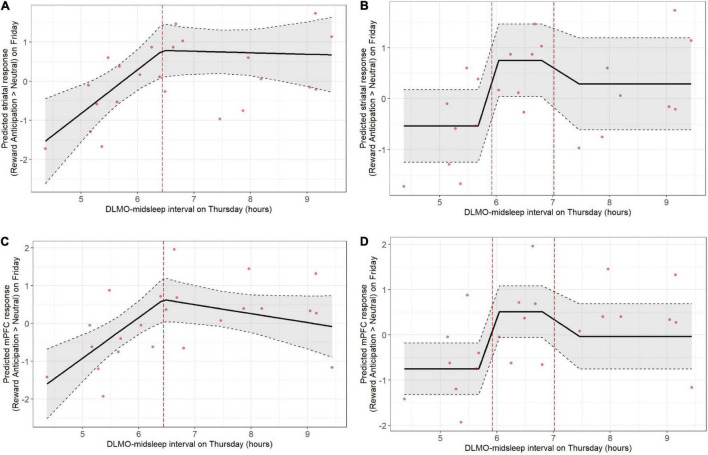
Weekday circadian alignment (DLMO-midsleep phase angle on Thursday) predicts pre-weekend neural response during anticipation of monetary reward (fMRI response on Friday), with shorter DLMO-midsleep intervals predicting lower neural responses. Findings were statistically significant, including after correction for multiple comparison, across both deviation (**A:** striatum, **C:** mPFC) and tertile (**B:** striatum, **D:** mPFC) models. Models accounted for age, sex, racial identity, and scan visit order.

Shorter DLMO-midsleep intervals on Thursday predicted lower neural responses during reward anticipation on Friday, across both striatum and mPFC, and based on both the deviation models and tertile models. In the deviation model (Figures 2A,C), a 1-h decrease in DLMO-midsleep interval (when below the median) was associated with a > 1 SD lower fMRI response (*b* = −1.12, *p* = 0.004 for striatal response, and *b* = −1.08, *p* = 0.002 for mPFC response). These findings were corroborated by tertile models (Figures 2B,D that indicated that having a shorter DLMO-midsleep interval (≤5.92 h) is associated with −1.28 SD reductions striatal activity (*p* = 0.012) and −1.26 SD reductions in mPFC activity (*p* = 0.003), relative to the medium group (<5.92 and ≤ 7.02-h). All of these findings survived multiple comparisons (Table 2 and Supplementary Table 10) (*post-hoc* contrasts indicated no significant differences between medium and long tertile groups).

**TABLE 2:** Weekday circadian alignment (Thursday DLMO-midsleep) predicting neural response (standardized score) during pre-weekend reward anticipation (Friday fMRI).

Pre-weekend reward anticipation		Deviation model				Tertile model^a^			
Striatal response	*Core model*	*b*	SE	*t*-value	*p*-value	*b*	SE	*t*-value	*p*-value
	Intercept	4.15	3.22	1.29	0.216	2.23	3.6	0.62	0.544
	Age	−0.16	0.14	−1.14	0.272	−0.1	0.16	−0.6	0.559
	Sex	−0.45	0.34	−1.33	0.201	−0.21	0.4	−0.52	0.613
	Racial identity	−0.11	0.26	−0.41	0.691	0.11	0.29	0.37	0.716
	Scan visit	0.64	0.36	1.81	0.089	0.51	0.39	1.32	0.206
	DLMO-midsleep - short	−1.12	0.33	−3.38	**0.004^c^**	−1.28	0.46	−2.82	**0.012^c^**
	DLMO-midsleep - long	0.04	0.19	0.20	0.848	−0.46	0.52	−0.89	0.389
	*Core model + prior night’s TST* ^b^								
	Thursday night TST	0.06	0.15	0.37	0.719	0.24	0.19	1.29	0.223
	DLMO-midsleep - short	−1.21	0.31	−3.94	**0.002^c^**	−1.60	0.49	−3.29	**0.006^c^**
	DLMO-midsleep - long	0.16	0.19	0.86	0.406	−0.93	0.59	−1.58	0.139
mPFC response	*Core model*								
	Intercept	0.32	2.74	0.12	0.908	−0.99	2.87	−0.35	0.734
	Age	−0.06	0.12	−0.48	0.636	−0.01	0.13	−0.12	0.91
	Sex	0.07	0.29	0.25	0.808	0.27	0.32	0.84	0.416
	Racial identity	0.46	0.22	2.08	0.054	0.59	0.24	2.53	0.022
	Scan visit	0.70	0.30	2.32	**0.034**	0.58	0.31	1.88	0.078
	DLMO-midsleep - short	−1.08	0.28	−3.82	**0.002^c^**	−1.26	0.36	−3.46	**0.003^c^**
	DLMO-midsleep - long	0.24	0.16	1.49	0.156	−0.54	0.41	−1.32	0.206
	*Core model + prior night’s TST* ^b^								
	Thursday night TST	−0.22	0.13	−1.71	0.113	−0.12	0.16	−0.74	0.47
	DLMO-midsleep - short	−1.04	0.27	−3.87	**0.002^c^**	−1.28	0.42	−3.08	**0.010^c^**
	DLMO-midsleep - long	0.21	0.17	1.24	0.238	−0.50	0.50	−0.99	0.344

*^a^“Medium” DLMO-midsleep was the referent group for Tertile Models. Thus, coefficient estimates for phase angle “DLMO-midsleep - short” and “DLMO-midsleep - long” indicate changes relative to the “medium” phase angle group, respectively.*

*^b^The model adding prior night’s total sleep time (TST) includes all the covariates included in the Core model, but they are not listed for clarity of presentation. All covariate effects remained non-significant in subsequent models.*

*^c^Outcome survived Benjamin-Hochberg multiple comparison correction. Bolded values indicate p < 0.05.*

However, Thursday DLMO-midsleep intervals that were *longer* than the median and/or in the long tertile (>7.02-h DLMO-midsleep interval) were not associated with neural responses to reward anticipation on Friday. All of these findings persisted after accounting for total sleep time on Thursday night.

In contrast, DLMO-midsleep on Thursday did not predict the neural response during reward outcome on Friday in either brain region, or in either deviation or tertile models (Supplementary Tables 1, 10).

### Primary Analyses – Does Pre-weekend Reward-Related Brain Function Predict Weekend Alcohol Use?

The neural response to reward anticipation on Friday, whether in striatum or mPFC, did not predict the number of drinks consumed over the subsequent weekend (Supplementary Tables 2, 10).

**FIGURE 3:**
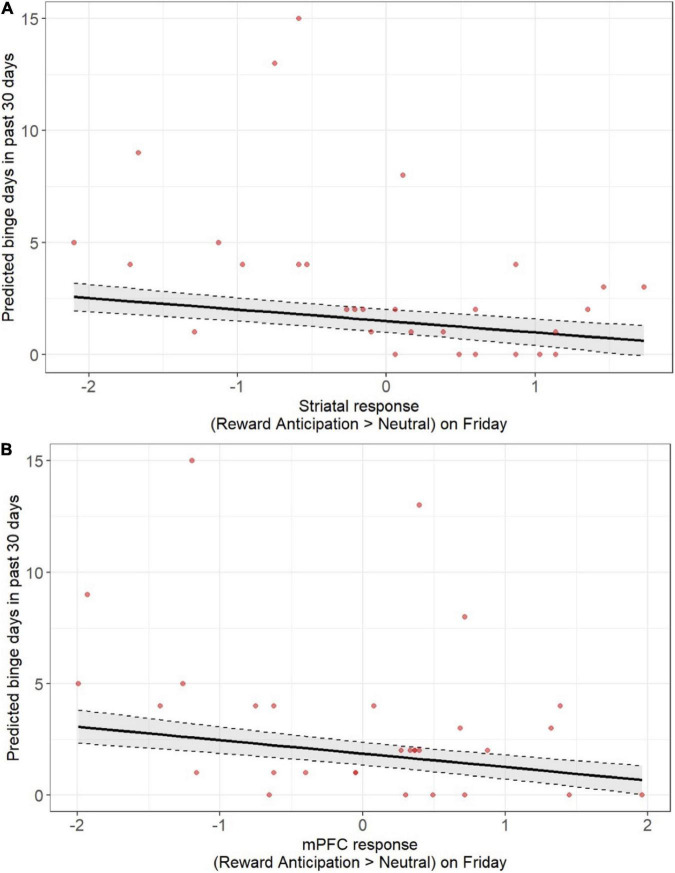
Lower pre-weekend neural responses during anticipation of monetary reward were associated with more days of being alcohol use over the past 30 days when assessed at screening. Findings were statistically significant, including after correction for multiple comparison, across both the striatum **(A)** and mPFC **(B)**. Models accounted for age, sex, racial identity, and scan visit order.

Given the smaller available sample with weekend alcohol data (*n* = 24) and the possibility that study procedures may have suppressed alcohol use during the study weekend, we also devised *post-hoc* analyses to examine whether the neural response to reward anticipation on Friday was associated with alcohol use during the screening at study entry. We focused on binge days over the past 30 days as a measure of problematic drinking. Smaller neural responses to reward anticipation on Friday, across both the striatum and mPFC, were associated with more binge days over 30 days (Figures 3A,B and Supplementary Tables 3, 10) after accounting for age, sex, racial identity, and scan visit order (striatum: β = 0.60, *p* < 0.001; mPFC: β = 0.55, *p* < 0.001), and survived multiple comparison correction.

### Secondary Analyses – Does Weekend Circadian Alignment Predict Post-weekend Reward-Related Brain Function?

The DLMO-midsleep intervals on Sunday were unrelated to the neural responses during reward anticipation (Supplementary Tables 4, 10) or outcome (Supplementary Tables 5, 10) on Monday, whether in the striatum or mPFC, and across both the deviation models and tertile models.

### Secondary Analyses – Does Social Jet Lag Predict Pre- or Post-weekend Reward-Related Brain Function?

**FIGURE 4:**
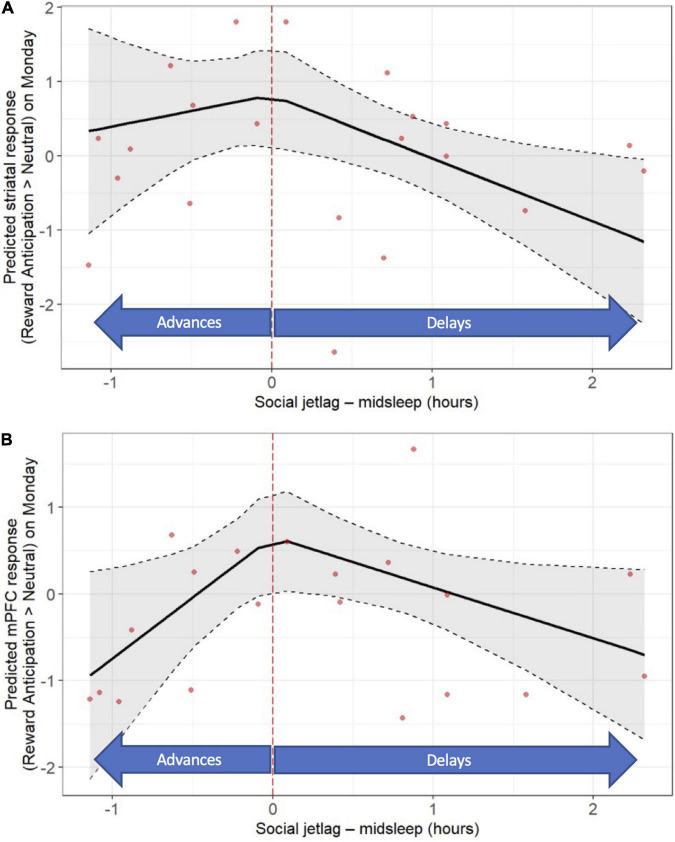
“Classic” social jet lag (weekday-weekend differences in actigraphy-based midsleep) is associated with post-weekend neural response during reward anticipation (fMRI response on Monday). Later midsleep on the weekend relative to weekday was associated with a lower striatal **(A)** response, and both later and earlier midsleep on the weekend were associated with a lower mPFC **(B)** response. Models accounted for age, sex, racial identity, and scan visit order.

With respect to “classic” social jet lag, *later* midsleep on the weekend relative to weekdays was associated with lower neural responses to reward anticipation on Monday, across both striatum (*b* = −0.85; *p* < 0.016) and mPFC (*b* = −0.59; *p* < 0.0049) (see Figures 4A,B and Supplementary Tables 6, 10). In addition, *earlier* midsleep on the weekend relative to weekdays was associated with a lower mPFC response to reward anticipation on Monday (*b* = 1.40, *p* = 0.029; Figure 4B and Supplementary Tables 6, 10). No significant associations between “classic” social jet lag and the neural response to reward outcome on Mondays were observed (Supplementary Tables 7, 10). No significant associations between “classic” social jet lag and the neural response to reward anticipation or outcome on Friday were observed (data not shown).

**FIGURE 5:**
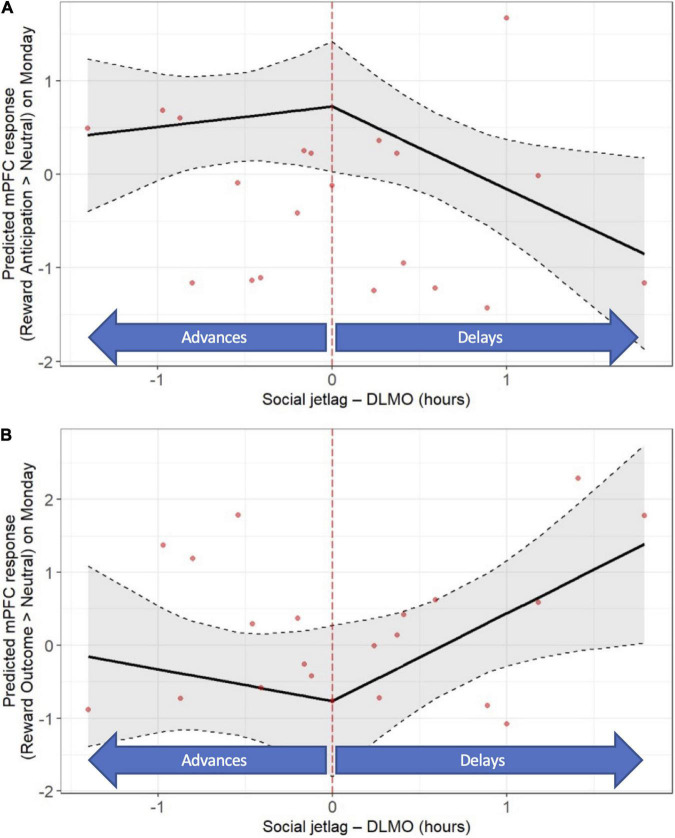
“Objective” social jet lag (weekday-weekend differences in DLMO) is associated with post-weekend neural response (fMRI response on Monday). Later DLMO on the weekend is associated with a lower mPFC response during reward anticipation **(A)** and a higher mPFC response to response during reward outcome **(B)**. Models accounted for age, sex, racial identity, and scan visit order.

With respect to “objective” social jet lag, *later* DLMO on Sunday relative to Thursday was associated with a lower mPFC response to reward anticipation on Monday (*b* = −0.88, *p* = 0.035; Figure 5A and Supplementary Tables 8, 10), as well as a higher mPFC response to reward outcome on Monday (*b* = 1.20, *p* = 0.036; Figure 5B and Supplementary Tables 9, 10). No significant associations between “objective” social jet lag and striatal response to reward anticipation or outcome on Mondays were observed (Supplementary Tables 8–10). No significant associations between “objective” social jet lag and neural response to reward anticipation or outcome on Friday were observed (data not shown). A summary of the findings from all the primary and secondary analyses is shown in Supplementary Table 10.

## Discussion

The present analyses examined whether circadian misalignment, measured *via* several objective indices, predicted reward-related brain function in a sample of late adolescent drinkers. Our primary findings were broadly consistent with hypotheses, showing that greater weekday circadian misalignment, as measured by a shorter DLMO-midsleep phase angle on Thursday, predicted lower pre-weekend (Friday) neural response to reward anticipation in the mPFC. In contrast to our original predictions based on data from similar-aged samples (Hasler et al., 2013, 2017a), circadian misalignment was associated with lower, not higher striatal response to reward, a pattern more consistent with findings from somewhat younger adolescents (Hasler et al., 2012, 2021). While the pre-weekend neural response to reward anticipation did not predict, in turn, subsequent weekend alcohol use assessed *via* EMA, it was associated with alcohol use history assessed at study entry. Specifically, lower striatal and mPFC responses to reward anticipation were both associated more frequent binge-level drinking over 30 days. We also observed more complex associations between our alternative social jet lag measures of circadian misalignment and reward-related brain function. In general, greater social jet lag (larger shifts in weekday-weekend sleep/circadian timing) was associated with lower neural response during reward anticipation on the Monday scan, across the striatum and mPFC, and most consistently when timing shifted later during the weekend. A notable exception was observed for social jet lag based on DLMO, for which weekend delays in DLMO were associated with *higher* mPFC responses during reward outcome on Monday. Altogether, our findings provide novel evidence that misalignment prospectively predicts proximal reward-related brain function, and further support the relevance of circadian misalignment to risk for alcohol use during late adolescence.

Our primary findings are consistent with the hypothesis that late adolescents experiencing circadian misalignment, specifically characterized by individuals attempting to sleep at an earlier biological time, exhibit an altered neural response during anticipation of rewards. Our prospective design allows for temporal precedence, thus providing additional credence that the circadian misalignment may be impacting the responsivity of neural circuitry. The finding held after accounting for total sleep time on the night prior to the scan, suggesting individual differences in sleep restriction were not driving the observed association. Furthermore, the lower responsivity in the mPFC is consistent with what we observed when experimentally imposing circadian misalignment in healthy 13–17 y/o’s (Hasler et al., 2021). Although only the findings for reward outcome met the corrected statistical threshold in the experimental study, findings for reward anticipation were in the same direction with a comparable effect size. The concordance with prior findings from similarly-aged samples is more mixed. In a sample of 20 y/o men with a range of alcohol use, evening-types [presumably experiencing more circadian misalignment; (Mongrain et al., 2006; Wittmann et al., 2006)] showed a relatively lower mPFC response during reward anticipation, which correlated in turn with alcohol consumption. This is consistent with the current findings. On the other hand, the evening-types also showed relatively *greater* ventral striatal response during reward outcome, which correlated in turn with greater symptoms of alcohol dependence. Furthermore, in a longitudinal analysis of a larger sample from that same study, eveningness (measured continuously) at age 20 was associated with *greater* response across both the ventral striatum and mPFC during reward outcome at age 22. This suggests that cross-sectional, short-term prospective, and long-term prospective relationships between circadian indices and reward response may diverge. In addition, prior work indicates that self-reported eveningness has complex relationships with circadian misalignment, with shorter DLMO-sleep phase angles only in those with more extreme delays (Mongrain et al., 2004). Indeed, in the present dataset, eveningness showed only a small, nonsignificant correlation (r = 0.21, *p* = 0.31) with DLMO-midsleep on Thursday (greater eveningness ∼ shorter DLMO-midsleep phase angles). Also, in contrast to prior findings, circadian alignment based on DLMO-midsleep did not predict the neural response to reward outcome, although the magnitude of the coefficients and, in one case, a trend-level *p*-value (Supplementary Table 1) suggest these relationships may achieve statistical significance in a larger sample. As all of these studies used variants of the same monetary reward task, we speculate that the inconsistencies in findings may relate to between-study differences in age, alcohol use history, and/or temporal relationship between the circadian and fMRI assessments. Finally, in the present sample, circadian alignment on Sunday was unrelated to reward-related brain response on Monday, and the mostly small effect sizes (Supplementary Tables 4, 5) suggest this was not simply due to insufficient statistical power.

The pre-weekend neural response to reward anticipation did not prospectively predict alcohol use on the subsequent weekend, but it was associated with history of binge-level drinking. Several explanations for these divergent relationships are possible. First, participating in the study, including visits to the sleep lab on Thursday and Sunday nights, may have suppressed typical alcohol use on Friday and Saturday nights, thereby obscuring any associations with the neural response would otherwise be present. In contrast, the drinking data collected at screening was available in more participants and may also provide a more reliable measure of habitual drinking. Alternatively, the association with the binge drinking at screening may reflect an effect of drinking history on reward circuitry—that binge drinking over time may lead to reduced neural responses to non-alcohol rewards, consistent with incentive-sensitization theory (Berridge and Robinson, 2016). This speculation is supported by evidence of decreased ventral striatal response during monetary reward in detoxified individuals with AUD (Wrase et al., 2007) and intravenous alcohol acutely decreased caudate activation during winning and losing trials (Gilman et al., 2012). However, other evidence suggests a positive association between binge history and nucleus accumbens response to reward (Crane et al., 2017), as well as greater nucleus accumbens response to monetary reward and greater wanting for alcohol after alcohol administration (Radoman et al., 2021). Furthermore, the binge drinking at screening may correlate with other trait-like processes associated with lower neural response to reward, perhaps reflecting “reward deficiency” that puts individuals at risk for alcohol problems (Bowirrat and Oscar-Berman, 2005).

Findings based on the social jet lag measures were more complex, although most were consistent with the hypothesis that greater misalignment, in this case as measured by larger delays in sleep/circadian timing from weekdays to weekends, is associated with lower reward-related brain responses. Findings were mostly with respect to reward anticipation, with larger weekday-weekend differences in midsleep correlating with lower striatal and mPFC responses, regardless of direction of shift (advances or delays). Larger delays in DLMO on weekends was also associated with lower mPFC response during reward anticipation on Monday. We did not observe any correlations between the social jet lag measures and with Friday reward scans, perhaps suggesting that the consequences of social jet lag are most notable at the start of the school/work week, when individuals need to abruptly advance their sleep schedules to meet school/work obligations on Monday morning. The findings were generally consistent with a prior study reporting that social jet lag characterized by larger weekend delays in midsleep was associated with lower striatal and mPFC response to monetary reward in healthy younger (age 12–14) adolescents (Hasler et al., 2012), although in that study, the associations were similar across anticipation and outcome phases of reward. Again, time of week may be relevant in understanding between-study differences; scans in the prior study occurred midweek rather than the end (Friday) or start (Monday) of the week in the present study.

The finding that larger DLMO delays on weekends were associated with *greater* mPFC response during reward outcome was unexpected, and without obvious explanation. We note that we also saw divergent findings depending on whether social jet leg was based on midsleep or DLMO in the prior manuscript (Hasler et al., 2019a), where weekend delays in midsleep were associated with greater weekend alcohol use, but DLMO shifts in either direction correlated with *lower* weekend alcohol use. It is important to note that social jet lag was conceived to capture misalignment by capturing the degree of mismatch between sleep timing on school/work days and sleep timing on free days, which should reflect innate circadian tendencies (Wittmann et al., 2006). Based on this definition, social jet lag and our primary measure of circadian misalignment (DLMO-midsleep) should correlate, but as noted in the prior manuscript (Hasler et al., 2019a), the social jet lag measures showed trivial and non-significant correlations with the DLMO-midsleep measure of circadian alignment. We speculate that social jet lag measures may better capture the instability and irregularity that can accompany circadian misalignment, that is *dynamic* aspects of misalignment, rather than the more precise but static snapshot of the DLMO-midsleep phase angle measure. Relatedly, recent findings suggest that delayed sleep/circadian timing overall, rather than social jet lag per se, may be more relevant to the dopaminergic aspects of reward function. In a sample of 32 healthy adults (middle-aged on average) that underwent positron emission tomography scans to measure dopamine receptor availability, Zhang et al. (2021) reported that delayed rest-activity rhythms, but not social jet leg (both based on wrist actigraphy), was associated with higher D1R availability in the caudate, as well as greater reported reward sensitivity to a methylphenidate administration. Interestingly, lower relative amplitude of the rest-activity rhythms (which could reflect misalignment) was associated with higher D2/D3R availability in the nucleus accumbens, which correlated in turn with reward sensitivity to methylphenidate.

In the present findings, we primarily found associations between our measures of circadian alignment and neural responses during reward anticipation, with only one instance of an association with response during reward outcome. We note that our prior studies examining associations between sleep/circadian characteristics and reward function have varied with respect to whether responses during anticipation, outcome, or both emerged as statistically significant findings (Hasler et al., 2012, 2017a,^2021^; Hasler and Clark, 2013), and we remain cautious in speculating about the nature of the apparently differential response here given the limitations of a small sample size. However, it is interesting to consider how the present findings most consistently support circadian misalignment predicting a smaller neural response during reward anticipation, which is in accordance with reward deficiency models of addiction risk. Our findings could also be seen as consistent with the incentive-sensitization theory, which would predict that individuals on the path to AUD would show diminishing anticipatory responses to non-alcohol rewards (like money), while anticipatory responses to alcohol rewards would increase and responses to reward outcome/receipt would remain unchanged (Berridge and Robinson, 2016). Future studies examining whether circadian alignment predicts alcohol-related reward would be useful for more comprehensive testing of the relevance to the incentive-sensitization theory. However, our data would seem to be at odds with neuroimaging studies linking increased striatal responses to reward with SUD risk (Heitzeg et al., 2015; Tervo-Clemmens et al., 2020). Also, while we have primarily focused on the relevance to addiction to this point, it may be worth considering how the present findings may be relevant to mood disorders, especially given the rich literature on associations between sleep/circadian characteristics, including circadian misalignment, and depression [e.g., (Emens et al., 2009; Hasler et al., 2010; Carpenter et al., 2021)]. Interestingly, growing neuroimaging evidence (including longitudinal data) links a decreased striatal response to reward to depression risk [e.g., (Fischer et al., 2019)] and the onset of depression [reviewed in Ng et al. (2019)]. Finally, evidence supports a combination of decreased striatal response and increased mPFC response to reward in the context of depression, suggesting that the mPFC may be dampening the striatal response (Forbes and Dahl, 2012), which may be relevant to interpreting our isolated finding of larger DLMO weekend delays relating to higher mPFC responses during reward outcome.

Although the present study has notable strengths, including objective measures of circadian alignment and a prospective design that allow consideration of temporal precedence, it also has several limitations worth mentioning. The sample is relatively small, particularly when considering all the key variables, thus limiting statistical power. Studies with larger sample sizes with sufficient statistical powered to conduct whole-brain analyses are needed to investigate the reward network beyond the striatum and mPFC and other relevant networks (e.g., control and salience networks). Larger samples will also permit consideration of potential sex or race differences in associations between circadian alignment and reward function, which seem plausible based on emerging data (e.g., (Hasler et al., 2019b; Hasler and Pedersen, 2020). While the design provided temporal precedence for testing the primary aims, it remains observational and cannot speak to causal relationships between circadian alignment, reward function, and alcohol use. In our deviation models, we arbitrarily selected the sample median in the DLMO-midsleep phase angle for use as the “normative” phase angle, but there is no consensus in the literature on ideal phase angle (we note this concern is somewhat mitigated by our parallel findings with the tertile models). Finally, a large portion of the participants were undergraduate students, with school, social, and sleep schedules that less systematically differ between weekdays and weekends. Thus, despite our efforts to capture circadian alignment on both weekdays and weekends, and the variability in sleep/circadian timing that occur, these measures may miss important day-to-day sleep/circadian instability that may be relatively more prominent in this population. Likewise, prospective studies in populations with more systematic weekday-weekend differences (e.g., high school students) might reveal more easily-interpretable social jet lag findings.

## Conclusion

Our findings provide preliminary evidence that objectively determined circadian misalignment on weekdays prospectively predicts pre-weekend reward-related brain function, which in turns is associated with habitual binge alcohol use. While replication in a larger sample is an important next step, the findings are broadly consistent with a growing literature suggesting that altered reward function may be a mechanistic link between sleep/circadian disturbances and alcohol involvement (Hasler and Clark, 2013; Logan et al., 2018). Although our recent manuscript supports a causal effect of circadian misalignment on reward-related brain function that is broadly consistent with the current findings, that study was in younger adolescents who were mostly substance-naive, and thus experimental designs are also needed in samples of substance-using individuals. Designs able to disentangle bidirectional relationships would be particularly useful, given clear evidence of alcohol effects on circadian rhythms (Meyrel et al., 2020). Future studies testing the effects of chronotherapeutic interventions on reward function would be informative, complementing observational research. Notably, if this work is collectively able to determine that circadian misalignment is contributing to altered reward function that increases the risk for problematic drinking, this has relevance to prevention and intervention efforts given that sleep and circadian rhythms are modifiable targets. Behavioral sleep interventions are efficacious at improving sleep in adults with AUD (Arnedt et al., 2011) and heavy-drinking late adolescents and young adults (Fucito et al., 2017; Miller et al., 2021). Although these studies have not demonstrated impact on drinking outcomes (compared to control conditions) to-date, methodological limitations may have masked such benefits (Miller et al., 2017), and none of these studies have included chronotherapeutic tools such as morning bright light, evening blue blockers, or evening melatonin, which would more directly target circadian misalignment. The promise of such interventions is further incentive for investment in further efforts to understand sleep/circadian-reward-alcohol relationships would be well justified.

## Data Availability Statement

The raw data supporting the conclusions of this article will be made available by the authors, without undue reservation.

## Ethics Statement

The studies involving human participants were reviewed and approved by Institutional Review Board of the University of Pittsburgh. The patients/participants provided their written informed consent to participate in this study.

## Author Contributions

BH conceived and designed the study and oversaw data collection. DC contributed to the study design. AS contributed to the scripting of the fMRI preprocessing scripts. BH, MW, and JG conceived and designed the analysis. MW and JG performed the analysis. BH, MW, JG, AS, and DC contributed to writing the manuscript. All authors contributed to the article and approved the submitted version.

## Conflict of Interest

The authors declare that the research was conducted in the absence of any commercial or financial relationships that could be construed as a potential conflict of interest. The reviewer JE declared past co-authorship with one of the authors BH to the handling Editor.

## Publisher’s Note

All claims expressed in this article are solely those of the authors and do not necessarily represent those of their affiliated organizations, or those of the publisher, the editors and the reviewers. Any product that may be evaluated in this article, or claim that may be made by its manufacturer, is not guaranteed or endorsed by the publisher.

## Footnotes

1
http://www.nitrc.org/projects/artifact_detect/

2
https://web.mit.edu/swg/software.htm
