# Supplementary material for: Preliminary Evidence That Circadian Alignment Predicts Neural Response to Monetary Reward in Late Adolescent Drinkers
Source: Front Neurosci. 2022 Feb 16;16:803349. doi: 10.3389/fnins.2022.803349 (PMC8888521; doi:10.3389/fnins.2022.803349)
Supplement: Supplementary file 1 [file Data_Sheet_1.docx]

**SUPPLEMENTAL MATERIALS**

**Supplemental Methods**

*fMRI Tasks*

Participants completed a total of two fMRI scans, counter-balanced to address potential task habituation effects (Plichta et al., 2012;Hasler et al., 2014). Participants were randomly assigned to either a Thursday/Friday-Sunday/Monday (‘FM’) or Sunday/Monday-Thursday/Friday (‘MF’) order.

Tasks were presented by projecting images onto a rear projection screen at the subject’s chest level and viewed through a mirror attached to the head coil. Stimulus presentation and registration of responses were controlled by a Windows-based computer running E-Prime 2.0 (Psychology Software Tools, 2012). Participants’ button-press responses were recorded using an RF-shielded response box and cable connected to the computer.

*Neuroimaging acquisition*

Neuroimaging was conducted on a Siemens Trio 3T scanner. Functional images were acquired using a simultaneous multi-slice (SMS) gradient echo EPI sequence and an oblique axial angle (17 interleaved slices acquired with SMS factor = 3 for a total of 51 slices, TR = 1500 ms, TE = 35  ms, Field of View (FOV) = 220 × 220  mm, matrix = 96 × 96, slice thickness = 2.3  mm, Flip Angle = 58°, Bandwidth = 1736 Hz/Px). In addition, Structural 3D axial MPRAGE images (TR = 1500 ms, TE = 3.19 ms, Flip Angle 8°, FOV = 256 × 256 mm, 1 mm isotropic voxels, 176 continuous slices) were acquired for co-registration and normalization of functional images.

**Neuroimaging preprocessing and analysis**

Neuroimaging data were preprocessed and analyzed using SPM12 (Ashburner et al., 2014). Preprocessing steps included realignment each participant’s data to the first volume in the time series to correct for head motion, coregistration of the realigned image with the subject’s anatomical image, segmentation of the structural scan to provide the necessary transformation parameters for spatial normalization, normalization of the functional scan to the standard Montreal Neurological Institute (MNI) template using the deformation field calculated during segmentation, and spatial smoothing with a Gaussian kernel of 6 mm full-width at half-maximum. Artifact Detection Toolbox (ART; http://www.nitrc.org/projects/artifact_detect/) software was used to detect functional volumes with signal intensity >4 SD from the subject's mean or >2mm composite interscan motion. Preprocessed data were inspected prior to second-level analysis to ensure that all scans had fewer than 20% of volumes with excessive movement detected by ART and good scan quality. Temporal censoring based on ART output was used to remove motion artifacts in first-level analysis for the remaining scans (Siegel et al., 2014). Two out of 33 available Friday scans and no Monday scans were excluded on this basis.

Preprocessed data were analyzed using a two-level random-effects procedure within a general linear modeling (GLM) framework. For each participant and scan (first-level), the main effect of task at each voxel was calculated for the relevant contrast of interest (e.g., Reward Anticipation>Neutral). Temporal censoring based on ART and the six movement parameters were included as regressors in order to control for participant movement. These first-level contrast images were then included in second-level 1-sample t-tests of each relevant contrast of interest: Reward Anticipation>Neutral and Reward Outcome>Neutral. We used the *rex* toolbox (<https://web.mit.edu/swg/software.htm>) to extract mean activations (p=1.0) across all voxels from *a priori* regions of interest (ROIs). For the Card Guessing Task, we employed bilateral striatum and the mPFC ROIs previously demonstrated to be responsive to the task. We focused on the caudate and putamen components of the striatum after determining that a majority of the scans had <80% coverage of the nucleus accumbens due to susceptibility artifacts. The bilateral caudate/putamen ROI was constructed using WFU PickAtlas Tool v2.4 and using the AAL atlas, consistent with prior studies examining sleep/circadian rhythms and using this task (Hasler et al., 2012;Hasler et al., 2013). The mPFC ROI was also constructed using the PickAtlas and defined as a 25-mm radius sphere centered on MNI coordinates x=0, y=44, z=22 and including medial Brodmann Areas (BA) 10 and BA32 (Forbes et al., 2010). All ROIs are shown in Figure S1. All scans had >80% coverage of the striatal and mPFC ROIs. Finally, we converted all extracted mean activations to z-scores in order to enhance interpretability.

Figure S1. The two region-of-interest (ROI) masks used in the study, including the bilateral striatum (caudate/putamen; **A**) and the medial prefrontal cortex (mPFC; **B**).

**B**

**A**


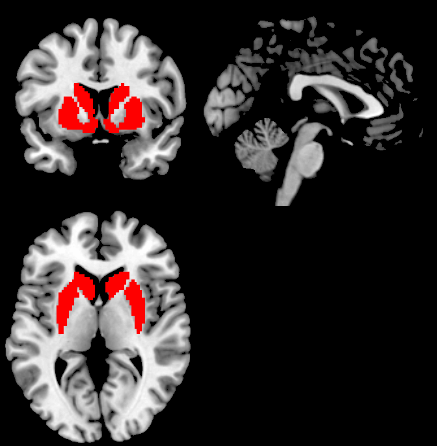

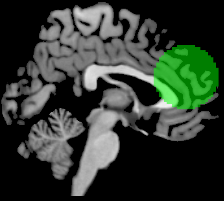


**Supplemental Results**

**Table S1** Weekday circadian alignment (Thursday DLMO-midsleep) predicting neural response (standardized score) during pre-weekend reward outcome (Friday fMRI)

|  |  |  | **Deviation Model** | | | |  | **Tertile Model** | | | |
| --- | --- | --- | --- | --- | --- | --- | --- | --- | --- | --- | --- |
|  | Parameter | | b | SE | t-value | p-value |  | b | SE | t-value | p-value |
| *Striatal* | Intercept | | -0.34 | 2.85 | -0.12 | 0.908 |  | 0.54 | 3.01 | 0.18 | 0.861 |
| *Response* | Age | | 0.05 | 0.13 | 0.38 | 0.708 |  | 0.02 | 0.14 | 0.15 | 0.879 |
|  | Sex | | 0.07 | 0.34 | 0.20 | 0.842 |  | -0.07 | 0.36 | -0.20 | 0.841 |
|  | Racial identity | | -0.30 | 0.24 | -1.28 | 0.219 |  | -0.39 | 0.25 | -1.57 | 0.136 |
|  | Scan Visit | | -0.14 | 0.36 | -0.38 | 0.712 |  | -0.05 | 0.36 | -0.15 | 0.886 |
|  | DLMO-midsleep - short | | 0.52 | 0.33 | 1.59 | 0.131 |  | 0.53 | 0.42 | 1.27 | 0.220 |
|  | DLMO-midsleep - long | | -0.14 | 0.19 | -0.76 | 0.458 |  | 0.42 | 0.48 | 0.86 | 0.399 |
| *mPFC* | Intercept | | 3.98 | 2.99 | 1.33 | 0.202 |  | 5.37 | 2.96 | 1.82 | 0.087 |
| *Response* | Age | | -0.12 | 0.14 | -0.89 | 0.386 |  | -0.17 | 0.14 | -1.29 | 0.214 |
|  | Sex | | 0.26 | 0.35 | 0.75 | 0.462 |  | 0.04 | 0.36 | 0.11 | 0.918 |
|  | Racial identity | | -0.64 | 0.25 | -2.55 | **0.021** |  | -0.78 | 0.25 | -3.13 | **0.006** |
|  | Scan Visit | | -0.21 | 0.38 | -0.55 | 0.591 |  | -0.13 | 0.36 | -0.38 | 0.710 |
|  | DLMO-midsleep - short | | 0.51 | 0.34 | 1.50 | 0.152 |  | 0.74 | 0.41 | 1.82 | 0.087 |
|  | DLMO-midsleep - long | | -0.23 | 0.20 | -1.15 | 0.265 |  | 0.85 | 0.47 | 1.79 | 0.091 |

**Table S2**. Pre-weekend neural response during reward anticipation predicting weekend alcohol use

|  | **Striatum** | | | |  | **mPFC** | | | |
| --- | --- | --- | --- | --- | --- | --- | --- | --- | --- |
| Parameter | IRR | SE | z-value | p-value |  | IRR | SE | z-value | p-value |
| Intercept | 0.00 | 2.21 | -2.62 | **0.009** |  | 0.01 | 2.12 | -2.41 | **0.016** |
| Age | 1.25 | 0.10 | 2.25 | **0.024** |  | 1.22 | 0.09 | 2.11 | **0.035** |
| Sex | 1.41 | 0.20 | 1.75 | 0.080 |  | 1.36 | 0.20 | 1.55 | 0.120 |
| Racial identity | 2.03 | 0.20 | 3.58 | **0.000** |  | 1.91 | 0.21 | 3.08 | **0.002** |
| Scan Visit | 0.98 | 0.19 | -0.12 | 0.908 |  | 0.95 | 0.19 | -0.25 | 0.801 |
| Mean ROI response | 1.12 | 0.12 | 0.94 | 0.347 |  | 1.11 | 0.12 | 0.93 | 0.352 |

NOTE: IRR=incidence rate ratio.

**Table S3**. Pre-weekend neural response during reward anticipation predicting number of binge days over past 30 days at study entry

|  | **Striatum** | | | |  | **mPFC** | | | |
| --- | --- | --- | --- | --- | --- | --- | --- | --- | --- |
| Parameter | β | SE | z-value | p-value |  | β | SE | z-value | p-value |
| Intercept | 0.08 | 2.21 | -1.12 | 0.261 |  | 0.01 | 2.44 | -2.06 | **0.039** |
| Age | 1.21 | 0.10 | 1.86 | 0.063 |  | 1.36 | 0.11 | 2.74 | **0.006** |
| Sex | 1.49 | 0.20 | 1.97 | **0.049** |  | 1.49 | 0.20 | 3.32 | **0.001** |
| Racial identity | 1.09 | 0.16 | 0.55 | 0.586 |  | 1.33 | 0.16 | 1.73 | 0.084 |
| Scan Visit | 0.87 | 0.21 | -0.63 | 0.531 |  | 1.02 | 0.22 | 0.11 | 0.914 |
| Mean ROI response | 0.60 | 0.11 | -4.65 | **0.000**^a^ |  | 0.55 | 0.12 | -4.91 | **0.000**^a^ |

**^a.^** Outcome survived Benjamin-Hochberg multiple comparison correction

**Table S4**. Weekend circadian alignment (Sunday DLMO-midsleep) predicting striatal response (standardized score) during post-weekend reward anticipation (Monday fMRI)

|  |  |  | **Deviation Model** | | | |  | **Tertile Model^a^** | | | |
| --- | --- | --- | --- | --- | --- | --- | --- | --- | --- | --- | --- |
|  | Parameter | | b | SE | t-value | p-value |  | b | SE | t-value | p-value |
| *Striatal* | Intercept | | 1.61 | 1.03 | 1.57 | 0.135 |  | 1.57 | 0.83 | 1.89 | 0.076 |
| *Response* | Age | | -0.06 | 0.04 | -1.60 | 0.128 |  | -0.06 | 0.04 | -1.62 | 0.123 |
|  | Sex | | -0.09 | 0.09 | -1.03 | 0.318 |  | -0.09 | 0.09 | -1.00 | 0.330 |
|  | Racial identity | | -0.10 | 0.06 | -1.64 | 0.120 |  | -0.11 | 0.06 | -1.76 | 0.096 |
|  | Scan Visit | | 0.18 | 0.10 | 1.88 | 0.078 |  | 0.18 | 0.10 | 1.79 | 0.091 |
|  | DLMO-midsleep - short | | 0.00 | 0.05 | -0.02 | 0.988 |  | 0.01 | 0.14 | 0.10 | 0.922 |
|  | DLMO-midsleep - long | | 0.00 | 0.04 | -0.11 | 0.912 |  | 0.00 | 0.12 | -0.03 | 0.975 |
| *mPFC* | Intercept | | 1.60 | 1.51 | 1.06 | 0.305 |  | 0.88 | 1.23 | 0.72 | 0.485 |
| *Response* | Age | | -0.07 | 0.06 | -1.15 | 0.268 |  | -0.05 | 0.06 | -0.95 | 0.359 |
|  | Sex | | -0.35 | 0.14 | -2.58 | **0.020** |  | -0.34 | 0.14 | -2.51 | **0.023** |
|  | Racial identity | | 0.06 | 0.10 | 0.57 | 0.575 |  | 0.08 | 0.09 | 0.88 | 0.390 |
|  | Scan Visit | | 0.08 | 0.15 | 0.53 | 0.605 |  | 0.05 | 0.15 | 0.35 | 0.733 |
|  | DLMO-midsleep - short | | -0.05 | 0.09 | -0.55 | 0.593 |  | 0.19 | 0.22 | 0.90 | 0.381 |
|  | DLMO-midsleep - long | | -0.03 | 0.06 | -0.54 | 0.597 |  | 0.11 | 0.17 | 0.62 | 0.546 |

^a.^ “Medium” DLMO-midsleep was the referent group for Tertile Models. Thus, coefficient estimates for phase angle “short” and “long” indicate changes relative to the “medium” phase angle group, respectively.

**Table S5.** Weekend circadian alignment (Sunday DLMO-midsleep) predicting neural response (standardized score) during post-weekend reward outcome (Monday fMRI)

|  |  |  | **Deviation Model** | | | |  | **Tertile Model^a^** | | | |
| --- | --- | --- | --- | --- | --- | --- | --- | --- | --- | --- | --- |
|  | Parameter | | b | SE | t-value | p-value |  | b | SE | t-value | p-value |
| *Striatal* | Intercept | | 0.89 | 1.58 | 0.56 | 0.582 |  | -0.01 | 1.29 | -0.01 | 0.996 |
| *Response* | Age | | -0.02 | 0.06 | -0.40 | 0.692 |  | -0.01 | 0.06 | -0.19 | 0.854 |
|  | Sex | | -0.22 | 0.14 | -1.62 | 0.124 |  | -0.21 | 0.15 | -1.46 | 0.163 |
|  | Racial identity | | 0.10 | 0.10 | 1.01 | 0.328 |  | 0.15 | 0.10 | 1.54 | 0.141 |
|  | Scan Visit | | -0.19 | 0.15 | -1.27 | 0.222 |  | -0.20 | 0.15 | -1.32 | 0.205 |
|  | DLMO-midsleep - short | | -0.07 | 0.08 | -0.83 | 0.416 |  | 0.18 | 0.21 | 0.83 | 0.417 |
|  | DLMO-midsleep - long | | -0.03 | 0.06 | -0.59 | 0.561 |  | 0.16 | 0.19 | 0.87 | 0.398 |
| *mPFC* | Intercept | | 0.82 | 4.41 | 0.19 | 0.854 |  | -1.50 | 3.51 | -0.43 | 0.675 |
| *Response* | Age | | 0.05 | 0.17 | 0.30 | 0.767 |  | 0.08 | 0.16 | 0.51 | 0.619 |
|  | Sex | | 0.04 | 0.39 | 0.12 | 0.910 |  | 0.04 | 0.40 | 0.09 | 0.931 |
|  | Racial identity | | -0.28 | 0.28 | -1.02 | 0.325 |  | -0.10 | 0.26 | -0.39 | 0.701 |
|  | Scan Visit | | -0.23 | 0.41 | -0.55 | 0.589 |  | -0.36 | 0.42 | -0.86 | 0.402 |
|  | DLMO-midsleep - short | | -0.15 | 0.23 | -0.66 | 0.519 |  | 0.78 | 0.58 | 1.36 | 0.193 |
|  | DLMO-midsleep - long | | -0.04 | 0.16 | -0.26 | 0.796 |  | 0.82 | 0.50 | 1.63 | 0.122 |

^a.^ “Medium” DLMO-midsleep was the referent group for Tertile Models. Thus, coefficient estimates for phase angle “short” and “long” indicate changes relative to the “medium” phase angle group, respectively.

**Table S6.** Social jet lag (Fri/Sat midsleep minus Tue/Wed midsleep) predicting post-weekend reward anticipation (Monday fMRI, standardized score)

|  | **Striatum** | | | |  | **mPFC** | | | |
| --- | --- | --- | --- | --- | --- | --- | --- | --- | --- |
| Parameter | b | SE | t-value | p-value |  | b | SE | t-value | p-value |
| *Core model* |  |  |  |  |  |  |  |  |  |
| Intercept | 14.45 | 3.88 | 3.72 | **0.002** |  | 4.72 | 3.47 | 1.36 | 0.191 |
| Age | -0.58 | 0.17 | -3.42 | **0.003** |  | -0.22 | 0.15 | -1.47 | 0.161 |
| Sex | -0.54 | 0.42 | -1.28 | 0.216 |  | -0.38 | 0.36 | -1.04 | 0.315 |
| Racial identity | -0.76 | 0.25 | -3.08 | **0.006** |  | 0.14 | 0.21 | 0.64 | 0.528 |
| Scan Visit | 1.10 | 0.37 | 2.95 | **0.009** |  | 0.67 | 0.33 | 2.05 | 0.057 |
| Social jet lag-DLMO-advance | 0.42 | 0.69 | 0.62 | 0.545 |  | 1.40 | 0.59 | 2.38 | **0.029** |
| Social jet lag-DLMO-delay | -0.85 | 0.32 | -2.65 | **0.016** |  | -0.59 | 0.28 | -2.12 | **0.049** |

**Table S7.** Social jet lag (Fri/Sat midsleep minus Tue/Wed midsleep) predicting post-weekend reward outcome (Monday fMRI, standardized score)

|  | **Striatum** | | | |  | **mPFC** | | | |
| --- | --- | --- | --- | --- | --- | --- | --- | --- | --- |
| Parameter | b | SE | t-value | p-value |  | b | SE | t-value | p-value |
| *Core model* |  |  |  |  |  |  |  |  |  |
| Intercept | -2.02 | 4.49 | -0.45 | 0.658 |  | -6.02 | 4.68 | -1.29 | 0.215 |
| Age | 0.04 | 0.20 | 0.23 | 0.820 |  | 0.25 | 0.20 | 1.24 | 0.231 |
| Sex | -0.87 | 0.49 | -1.79 | 0.090 |  | -0.32 | 0.51 | -0.62 | 0.541 |
| Racial identity | 0.56 | 0.28 | 1.97 | 0.064 |  | 0.20 | 0.30 | 0.69 | 0.496 |
| Scan Visit | -0.80 | 0.43 | -1.86 | 0.079 |  | -0.69 | 0.45 | -1.53 | 0.144 |
| Social jet lag-DLMO-advance | -0.80 | 0.79 | -1.01 | 0.327 |  | -1.25 | 0.83 | -1.52 | 0.146 |
| Social jet lag-DLMO-delay | 0.51 | 0.37 | 1.39 | 0.182 |  | 0.71 | 0.39 | 1.85 | 0.081 |

**Table S8.** Social jet lag (Sunday DLMO minus Thursday DLMO) predicting post-weekend reward anticipation (Monday fMRI, standardized score)

|  | **Striatum** | | | |  | **mPFC** | | | |
| --- | --- | --- | --- | --- | --- | --- | --- | --- | --- |
| Parameter | b | SE | t-value | p-value |  | b | SE | t-value | p-value |
| *Core model* |  |  |  |  |  |  |  |  |  |
| Intercept | 6.96 | 3.19 | 2.18 | 0.043 |  | 3.67 | 2.65 | 1.38 | 0.184 |
| Age | -0.27 | 0.14 | -1.89 | 0.076 |  | -0.15 | 0.12 | -1.24 | 0.231 |
| Sex | -0.52 | 0.41 | -1.29 | 0.213 |  | -1.47 | 0.33 | -4.51 | **0.000** |
| Racial identity | -0.58 | 0.25 | -2.35 | **0.030** |  | -0.03 | 0.20 | -0.13 | 0.895 |
| Scan Visit | 0.86 | 0.38 | 2.24 | **0.038** |  | 0.58 | 0.31 | 1.85 | 0.082 |
| Social jet lag-DLMO-advance | -0.82 | 0.50 | -1.65 | 0.117 |  | 0.22 | 0.40 | 0.54 | 0.596 |
| Social jet lag-DLMO-delay | -0.42 | 0.44 | -0.96 | 0.349 |  | -0.88 | 0.38 | -2.29 | **0.035** |

**Table S9.** Social jet lag (Sunday DLMO minus Thursday DLMO) predicting post-weekend reward outcome (Monday fMRI, standardized score)

|  | **Striatum** | | | |  | **mPFC** | | | |
| --- | --- | --- | --- | --- | --- | --- | --- | --- | --- |
| Parameter | b | SE | t-value | p-value |  | b | SE | t-value | p-value |
| *Core model* |  |  |  |  |  |  |  |  |  |
| Intercept | 2.37 | 4.31 | 0.55 | 0.589 |  | -0.26 | 3.89 | -0.07 | 0.948 |
| Age | -0.14 | 0.20 | -0.71 | 0.487 |  | -0.01 | 0.18 | -0.04 | 0.971 |
| Sex | -0.39 | 0.55 | -0.72 | 0.482 |  | 0.82 | 0.49 | 1.66 | 0.115 |
| Racial identity | 0.32 | 0.33 | 0.96 | 0.350 |  | -0.11 | 0.30 | -0.38 | 0.708 |
| Scan Visit | -0.16 | 0.52 | -0.30 | 0.765 |  | -0.15 | 0.47 | -0.33 | 0.748 |
| Social jet lag-DLMO-advance | 0.33 | 0.67 | 0.50 | 0.625 |  | -0.44 | 0.61 | -0.72 | 0.482 |
| Social jet lag-DLMO-delay | 0.26 | 0.59 | 0.44 | 0.668 |  | 1.20 | 0.53 | 2.26 | **0.036** |

**Table S10**. Summary of study findings: Associations between circadian alignment, alcohol use, and fMRI monetary reward variables.

|  | Friday scan | | | |  | Monday scan | | | |
| --- | --- | --- | --- | --- | --- | --- | --- | --- | --- |
|  | Reward anticipation | | Reward outcome | |  | Reward anticipation | | Reward outcome | |
|  | Striatum | mPFC | Striatum | mPFC |  | Striatum | mPFC | Striatum | mPFC |
| **DLMO-midsleep (Thursday) -- Primary measure** |  |  |  |  |  |  |  |  |  |
| Shorter phase angle | **lower**; p=0.004 | **lower**; p=0.002 | ns | ns |  | Not tested | | | |
| Longer phase angle | ns | ns | ns | ns |  | Not tested | | | |
| DLMO-midsleep (Sunday) |  |  |  |  |  |  |  |  |  |
| Shorter phase angle | Not tested | | | |  | ns | ns | ns | ns |
| Longer phase angle | Not tested | | | |  | ns | ns | ns | ns |
| "Classic" social jet lag (Sat/Sun-Tue/Wed midsleep) |  |  |  |  |  |  |  |  |  |
| Delaying (later midsleep on weekend) | **lower**; 0.004 | ns | ns | ns |  | **lower**; p=0.016 | **lower**; p=0.049 | ns | higher; p=0.081 |
| Advancing (earlier midsleep on weekend) | ns | ns | ns | ns |  | ns | **lower**; p=0.029 | ns | ns |
| Objective social jet lag (Sun-Thu DLMO) |  |  |  |  |  |  |  |  |  |
| Delaying (later DLMO on weekend) | ns | ns | ns | ns |  | ns | **lower**; p=0.035 | ns | **higher**; p=0.036 |
| Advancing (earlier DLMO on weekend) | ns | ns | lower; p=0.094 | ns |  | ns | ns | ns | ns |
| Weekend alcohol use (# of drinks) | ns | ns | Not tested | | | | | | |
| Binge alcohol use at screening (# of binges in past 30 days) | **lower**; p<0.001 | **lower**; p<0.001 | Not tested | | | | | | |
|  |  |  |  |  |  |  |  |  |  |

NOTES: Direction of reward response in bold when p<0.05; ns=p>0.10. Results for DLMO-midsleep measures all based on deviation models (not tertile models).

**REFERENCES**

Ashburner, J., Barnes, G., Chen, C., Daunizeau, J., Flandin, G., Friston, K., Kiebel, S., Kilner, J., Litvak, V., and Moran, R. (2014). SPM12 manual. *Wellcome Trust Centre for Neuroimaging, London, UK*.

Forbes, E.E., Ryan, N.D., Phillips, M.L., Manuck, S.B., Worthman, C.M., Moyles, D.L., Tarr, J.A., Sciarrillo, S.R., and Dahl, R.E. (2010). Healthy adolescents' neural response to reward: associations with puberty, positive affect, and depressive symptoms. *Journal of the American Academy of Child and Adolescent Psychiatry* 49**,** 162-172 e161-165.

Hasler, B.P., Dahl, R.E., Holm, S.M., Jakubcak, J.L., Ryan, N.D., Silk, J.S., Phillips, M.L., and Forbes, E.E. (2012). Weekend-weekday advances in sleep timing are associated with altered reward-related brain function in healthy adolescents. *Biological Psychology* 91**,** 334-341.

Hasler, B.P., Forbes, E.E., and Franzen, P.L. (2014). Time-of-day differences and short-term stability of the neural response to monetary reward: a pilot study. *Psychiatry Research: Neuroimaging* 224**,** 22-27.

Hasler, B.P., Sitnick, S.L., Shaw, D.S., and Forbes, E.E. (2013). An altered neural response to reward may contribute to alcohol problems among late adolescents with an evening chronotype. *Psychiatry Research: Neuroimaging* 214**,** 357-364.

Plichta, M.M., Schwarz, A.J., Grimm, O., Morgen, K., Mier, D., Haddad, L., Gerdes, A.B., Sauer, C., Tost, H., Esslinger, C., Colman, P., Wilson, F., Kirsch, P., and Meyer-Lindenberg, A. (2012). Test-retest reliability of evoked BOLD signals from a cognitive-emotive fMRI test battery. *NeuroImage* 60**,** 1746-1758.

Psychology Software Tools, I. (2012). "E-Prime 2.0". (Pittsburgh, PA: Psychology Software Tools, Inc.).

Siegel, J.S., Power, J.D., Dubis, J.W., Vogel, A.C., Church, J.A., Schlaggar, B.L., and Petersen, S.E. (2014). Statistical improvements in functional magnetic resonance imaging analyses produced by censoring high‐motion data points. *Human brain mapping* 35**,** 1981-1996.
